# Beyond cumulative impact: the he-index and a volume-normalized efficiency metric for research evaluation

**DOI:** 10.3389/frma.2026.1793664

**Published:** 2026-05-04

**Authors:** Mohamd Laimon

**Affiliations:** Department of Mechanical Engineering, Al-Hussein Bin Talal University, Ma'an, Jordan

**Keywords:** bibliometrics, field-weighted citation impact (FWCI), h-index, impact concentration, scientometrics

## Abstract

Traditional bibliometric indicators such as the h-index emphasize cumulative citation impact but provide limited insight into the efficiency and concentration of that impact across a researcher's publication portfolio. This study introduces the he-index as an interpretable indicator of the proportion of publications represented by the h-index core, relative to the researcher's total publication output. Using publicly accessible Scopus data from 54 researchers across engineering, medicine, and social sciences in the United States, China, and Australia, we examine the behavior of the he-index in relation to publication volume, career stage, and disciplinary context. As expected for a ratio-based indicator, the he-index exhibits a strong negative association with total publications (Spearman ρ = −0.77, *p* < 0.001), which may reflect structural sensitivity to publication volume. To improve cross-volume interpretability, we further propose a volume-normalized efficiency metric (he^*^ = *h*/ĥ), where the expected h-index is estimated using a power-law scaling model (ĥ = 1.02*N*^0.7^, *R*^2^ ≈ 0.87). The normalized metric shows no significant dependence on publication volume (ρ = 0.064, *p* = 0.645) and exhibits weaker career-stage sensitivity, while discipline-level differences remain non-significant. External validation using field-weighted citation impact (FWCI) available for a subset of researchers provides additional support for the normalized metric, with he^*^ positively associated with FWCI (ρ = 0.419, *p* = 0.003), whereas the raw he-index shows no significant association. Overall, the findings indicate that impact concentration and volume-normalized citation efficiency offer complementary perspectives to cumulative impact metrics, supporting more nuanced and multidimensional research evaluation.

## 1 Introduction

In academic and research-intensive environments, quantitative indicators are widely used to support decisions related to hiring, promotion, funding, and institutional benchmarking (Masic, 2024). Among author-level metrics, the h-index is one of the most influential and commonly reported indicators because it provides a single-value summary of a scholar's output while partially combining publication volume with citation impact (Ain et al., 2019). Since it was introduced by Hirsch (2005), the h-index has been adopted by major bibliographic databases and is frequently used in research assessment frameworks due to its simplicity and intuitive interpretation.

The literature on the h-index has evolved along two main directions: (i) critical examination of its limitations, including issues related to interpretability, disciplinary variation, and co-authorship effects, and (ii) the development of modified or complementary indicators addressing aspects such as career length, collaboration structure, and citation normalization (Ding et al., 2020; Mondal et al., 2023). Despite these developments, most existing indicators remain focused on cumulative impact or normalization dimensions, while comparatively limited attention has been given to explicitly characterizing how impact is distributed across a researcher's publication portfolio. Most prominently, it compresses a complex publication and citation profile into a single number and therefore cannot fully reflect the diversity of scholarly trajectories, publication practices, or citation dynamics across fields, institutions, and career stages (Ding et al., 2020; Masic, 2024). An important implication is that the h-index does not explicitly indicate how concentrated or distributed impact is across a researcher's overall publication portfolio. While the h-index effectively captures cumulative impact, it does not describe how that impact is distributed across individual publications. For example, two researchers may have the same h-index, yet one may have achieved this value through a compact set of consistently cited papers, while the other may have accumulated a larger number of publications with only a subset contributing meaningfully to the *h*-core. In both cases, the h-index reflects comparable overall impact, but does not reveal differences in the internal structure or concentration of that impact. Thus, identical h-index values may correspond to different underlying structures of scholarly influence, an aspect increasingly discussed in recent studies on the interpretability of bibliometric indicators (de Souza et al., 2025; Mondal et al., 2023). Accordingly, the objective of this study is to introduce a complementary indicator that captures the structural characteristics of research impact, rather than to replace or critique the h-index as a measure of cumulative performance.

The growing recognition of these limitations has motivated the development of numerous alternative author-level indicators. A comprehensive review by Wildgaard et al. (2014) identified more than 100 bibliometric indices proposed to evaluate individual researchers, many of which emphasize either output volume, cumulative citations, or the influence of a small subset of highly cited papers. However, many proposed metrics remain difficult to interpret, computationally complex, or insufficiently transparent for routine use in evaluation contexts. This highlights an ongoing need for simple and interpretable complementary measures that can provide additional insight beyond cumulative impact alone.

Motivated by this gap, the present study introduces a complementary metric referred to as the *h*-efficiency index (he-index). The he-index is intended to provide an interpretable description of impact concentration by quantifying the proportion of publications that contribute to a researcher's h-index. In contrast to cumulative indicators that primarily reflect total achievement, the he-index emphasizes impact density (i.e., the extent to which measurable influence is concentrated within a researcher's total output). Such context-sensitive perspectives have been repeatedly highlighted as important in bibliometric research, particularly when interpreting metrics across disciplines and career stages (Bornmann and Daniel, 2008; Joshi, 2014; Waltman, 2016).

Recent bibliometric research has continued to examine the limitations of cumulative citation indicators and the need for more context-sensitive approaches to research evaluation. Several studies have emphasized the importance of normalization strategies that account for structural differences in publication portfolios, citation practices, and collaboration patterns (Bornmann and Tekles, 2021; Ryazanova, 2022). In particular, recent discussions in scientometrics have highlighted the importance of developing complementary indicators that capture different dimensions of scholarly performance rather than relying on a single cumulative metric (Bihari et al., 2023; Ioannidis et al., 2024; Mondal et al., 2023). These developments reinforce the need for simple and interpretable indicators that can reveal structural characteristics of publication portfolios, including the relationship between publication volume and the concentration of impactful publications. The proposed he-index and its normalized form he^*^ contribute to this line of research by focusing specifically on the representation and efficiency of impactful publications relative to overall output.

Several extensions of the h-index have been proposed to address specific limitations of cumulative impact metrics. For example, the *m*-index normalizes the h-index by career length, allowing comparisons across researchers at different stages of their academic trajectory (Hirsch, 2005). Other variants adjust for co-authorship contributions through fractional counting approaches, such as the *hm*-index (Schreiber, 2008), while field-normalized indicators such as field-weighted citation impact (FWCI) account for disciplinary citation differences in citation practices (Ioannidis et al., 2020). These approaches primarily focus on temporal normalization, authorship contribution, or field variation. However, they do not explicitly address the relationship between publication volume and the concentration of impactful publications within a researcher's output. The proposed he-index and its normalized form (he^*^) address this gap by examining the proportion of a researcher's publication portfolio represented by the h-index core and by evaluating citation impact relative to expected scaling with publication volume.

To further illustrate the practical relevance of distinguishing between cumulative impact and impact structure, consider the following example. Two researchers may have identical h-index values but different publication profiles: one may achieve this value through a relatively small set of consistently cited publications, while another may reach the same h-index through a larger body of work with a more uneven citation distribution. Although both profiles yield the same cumulative indicator, they reflect different patterns of scholarly influence. In practical evaluation contexts such as academic hiring, promotion, or funding decisions, such differences may influence how research performance is interpreted, particularly when evaluators seek to distinguish between concentrated and dispersed impact profiles. The proposed he-index and its normalized form (he^*^) aim to make this structural dimension more explicit and interpretable.

### 1.1 Theoretical foundation and metric definition

The h-index identifies the largest number *h* such that a researcher has at least *h* papers each cited *h* times (Hirsch, 2005). While this definition provides a useful summary of cumulative impact, it does not explicitly capture the relationship between the *h*-core and the researcher's full publication output. Specifically, the h-index does not indicate how many total publications were required to reach a given *h*, nor does it reveal how large the set of contributing publications is relative to overall output.

To address this interpretive limitation, we define the he-index as:


$$\begin{array}{l} he=\left(\frac{h}{N}\right)\; \end{array}$$

where *h* is the h-index and *N* is the total number of publications. The he-index expresses the percentage of a researcher's publications represented by the h-index core relative to the total publication output, providing an interpretable indication of how the h-index relates to the overall publication portfolio. A higher he-index indicates that a larger fraction of output contributes to the *h*-core, implying a more concentrated impact profile; conversely, a lower value indicates that measurable impact is concentrated within a smaller subset of publications relative to the total volume.

It is important to emphasize that the he-index is not intended to replace established bibliometric indicators. Rather, it is designed as a complementary measure that highlights a dimension of performance not directly captured by cumulative metrics. Conceptually, the he-index differs from commonly used alternatives. For instance, the m-index normalizes the h-index by academic age (years since the first publication) to account for time-based accumulation, whereas the he-index normalizes the h-index by publication volume, focusing on portfolio concentration rather than career length. Similarly, the *g*-index gives additional weight to highly cited publications and therefore reflects citation intensity among top papers, while the he-index captures how broadly a researcher's output contributes to the *h*-core. In this way, the he-index provides an interpretable measure of impact density, which may be particularly informative when comparing researchers with substantially different publication volumes.

At the same time, because *he* = *h*/*N* is a ratio-based indicator, it is intrinsically sensitive to publication count and may systematically decline as publication portfolios expand. For this reason, the he-index should be interpreted carefully, especially when comparing researchers across substantially different career stages or output scales. To enable more meaningful cross-volume comparison, this study further introduces a volume-normalized efficiency score that benchmarks observed h-index values against the expected h-index conditional on publication count. Specifically, the expected h-index ĥ is estimated using a power-law scaling model of the form:


$$\begin{array}{l} ĥ=aN^{b}\; \end{array}$$

And a normalized efficiency index is computed as:


$$\begin{array}{l} he^{*}=\frac{h}{ĥ}\; \end{array}$$

This normalized metric reduces the structural dependence on publication volume and supports interpretation of citation efficiency relative to typical performance at comparable output levels.

To situate the proposed indicators within the broader landscape of author-level bibliometric metrics, Table 1 presents a structured comparison between the he-index and several widely used indicators, including the h-index, the *m*-index, the hm-index, the output-normalized score (OnS), and the composite citation indicator. These metrics differ in the dimensions they attempt to normalize, including career length, co-authorship structure, field-specific citation practices, and publication volume. The comparison highlights how the proposed he-index and its normalized form he^*^ relate conceptually to existing author-level indicators.

**Table 1:** Conceptual comparison of the proposed indicators with established author-level bibliometric metrics.

Indicator	Concept	What it measures	Normalization dimension	Data requirement	Key sensitivity
h-index (Hirsch, 2005)	Citation threshold	Cumulative citation impact	None	Author-level citation counts	Favors large publication portfolios
m-index (Hirsch, 2005)	*h*/*years*	Impact adjusted for career length	Career time	Publication year of first paper	Sensitive to career stage
hm-index (Schreiber, 2008)	Fractional h-index	Impact adjusted for co-authorship	Co-authorship	Document-level authorship counts	Sensitive to collaboration size
Output-normalized score (OnS) (Onjia, 2025)	Citation score normalized by output	Balance between productivity and citations	Publication output	Document-level citation distribution	Sensitive to citation distribution
Composite indicator (ci) (Ioannidis et al., 2020)	Weighted combination of citation indicators	Multidimensional citation influence	Field and citation structure	Detailed document-level citation metrics	Complex citation patterns
he-index (this study)	*h*/*N*	Proportion of publications represented by the h-core	Publication volume	Author-level publication and h-index data	Decreases with increasing output
he^*^ (this study)	*h*/ĥ	h-index performance relative to expected output	Publication-volume scaling	Author-level publication and h-index data	Reduces output-size bias

As shown in Table 1, existing indicators primarily normalize citation impact with respect to career length, co-authorship structure, or field variation. In contrast, the proposed he-index and its normalized form he^*^ examine the relationship between publication volume and the representation or efficiency of impactful publications within a researcher's output. A full empirical comparison with indicators such as the hm-index, output-normalized score (OnS), and the composite citation indicator proposed by Ioannidis et al. (2020) would require detailed document-level bibliometric data, including co-authorship counts, citation distributions, and field-normalized citation parameters for individual publications. Because the present study relies on aggregated author-level profile data extracted from Scopus, such variables were beyond the scope of the available dataset.

Therefore, Table 1 provides a conceptual comparison highlighting the structural properties, normalization targets, and sensitivities of these indicators relative to the proposed metrics. This comparison situates the he-index and he^*^ within the broader landscape of bibliometric indicators and clarifies their distinct analytical focus. In particular, the normalized indicator he^*^ introduces a volume-adjusted perspective by comparing the observed h-index with the expected h-index derived from publication-output scaling, allowing researchers with different publication volumes to be evaluated on a comparable efficiency basis. Importantly, he^*^ provides additional discriminative capacity by comparing the observed h-index with the expected value derived from publication volume, allowing researchers with similar h-index values but different publication outputs to be evaluated relative to expected performance.

### 1.2 Study objectives and contributions

Accordingly, the aim of this paper is to introduce and examine the he-index as an interpretable measure of impact concentration, and to evaluate its behavior across researchers from different disciplines and career stages using Scopus-derived bibliometric data. The contributions of this study are fourfold: (1) proposing a transparent concentration-based metric derived from the h-index, (2) empirically demonstrating its structural sensitivity to publication volume, (3) introducing a volume-normalized efficiency score to improve cross-scale interpretability, and (4) providing comparative statistical analysis across discipline and career-stage groups to support the use of efficiency, and concentration-oriented indicators as complements to cumulative impact measures.

In practical evaluation contexts, cumulative indicators such as the h-index may assign similar scores to researchers with substantially different publication profiles, limiting the ability to distinguish between concentrated and dispersed impact structures. The proposed he-index and its normalized form address this limitation by providing additional insight into how impact is distributed across a researcher's output and whether citation performance exceeds expectations given publication volume. This enables more nuanced interpretation of scholarly performance in hiring, promotion, and benchmarking contexts.

## 2 Methodology

This study adopts a quantitative bibliometric approach combining theoretical formulation with empirical evaluation to examine the interpretability and statistical behavior of the proposed he-index. A dataset was compiled from publicly accessible Scopus author profiles, capturing diversity across research disciplines, geographical contexts, and academic career stages. The overall objective is to define the proposed indicator, compute it using real bibliometric profiles, and assess its behavior across groups and in relation to publication volume.

Bibliometric data were collected from publicly accessible Scopus author profiles using the Scopus web interface. For each researcher, the following fields were recorded: total publication count, h-index, and FWCI when available. Data were manually compiled into a structured dataset for analysis. Statistical analyses were conducted using Python-based statistical routines for regression fitting, correlation analysis, and non-parametric statistical tests. Spearman rank correlation coefficients were used to assess associations between bibliometric indicators due to the non-normal distribution of the data. FWCI values were obtained from the author-level portfolio indicator displayed in the Scopus interface, representing the average field-weighted citation impact across the publications associated with a given author profile.

### 2.1 Data source and sampling design

Data were collected from publicly available Scopus author profiles, which provide author-level bibliometric indicators including the total number of indexed publications and h-index values. A total of 54 researchers were included in the sample to ensure structured diversity across three disciplinary domains (engineering, medicine, and social sciences) and three regions (United States, China, and Australia). Researchers were selected using a stratified random sampling approach from publicly available Scopus author profiles, with predefined criteria to ensure variation in publication volume, h-index values, disciplines, and geographic regions.

The selection of researchers from the United States, China, and Australia was intended to provide representation from major research systems across different global regions. These countries collectively account for a substantial share of global scientific output and citation activity, as reported in large-scale international assessments of research performance (Commission, 2024; Lewis et al., 2021), allowing the empirical illustration of the proposed metric across diverse research environments. The sample was designed to demonstrate the methodological behavior of the metric rather than to represent a globally exhaustive dataset. All bibliometric data were extracted from publicly accessible Scopus author profiles. Data extraction was conducted in July 2025, and all reported metrics correspond to the values available at that time. For each researcher, the following information was recorded: total publications (*N*), h-index (*h*), discipline, region, and career-stage category.

To ensure data integrity, basic quality-control checks were applied during data collection. Author profiles were visually inspected to confirm correct author identification and to minimize potential disambiguation issues associated with common names. Profiles showing clear evidence of duplication or incomplete publication records were excluded. Because Scopus author profiles already aggregate publications attributed to an individual researcher, document-level filtering was not applied; instead, the analysis relied on the summary metrics reported at the author-profile level. These steps were implemented to reduce potential errors associated with author-profile duplication or misattribution. The anonymized dataset and diagnostic visualizations used in the analysis are provided in the Supplementary Table A1 and Supplementary Figures A1–A4.

### 2.2 Career-stage classification

For comparative analysis, researchers were categorized into early-career, mid-career, and senior groups based on bibliometric benchmarking practices reported in prior research assessment studies (Ain et al., 2019; Bensman, 2011; Bornmann and Daniel, 2009). Specifically, researchers were classified using h-index thresholds as follows: early-career (*h* ≤ 10), mid-career (11 ≤ *h* ≤ 30), and senior (*h* > 30). These thresholds are used as practical bibliometric benchmarks to approximate differences in publication portfolios when detailed career timeline information (such as years since first publication) is unavailable. The grouping therefore serves as a descriptive analytical device rather than a strict representation of academic career stages. Because the he-index is mathematically derived from the h-index, career-stage classification is treated as a descriptive grouping variable, and results are interpreted cautiously to avoid circularity in evaluation. In particular, differences across career stages are not presented as validation of research quality, but as an analysis of how impact concentration metrics behave across publication portfolio structures.

### 2.3 Metric formulation

#### 2.3.1 he-index (h-core proportion indicator)

The h-index is defined as the largest number *h* such that a researcher has published at least *h* papers, each cited at least *h* times. While widely used as a cumulative measure of academic influence, the h-index does not explicitly indicate how concentrated this influence is within the overall publication portfolio. To address this interpretive gap, the he-index is defined as:


$$\begin{array}{l} he=\left(\frac{h}{N}\right)\; \end{array}$$

where *h* is the h-index and *N* is the total number of publications. The he-index can be interpreted as the percentage of publications contributing to the *h*-core, thereby providing a transparent measure of impact concentration (impact density) rather than total output.

Unlike the h-index, which summarizes cumulative performance, the he-index highlights whether impact is broadly distributed across a scholar's output or concentrated within a smaller subset of publications. This interpretation differs from other commonly used extensions: for example, the *m*-index adjusts the h-index by academic age, while the *g*-index emphasizes highly cited papers. In contrast, the he-index focuses on the ratio between the *h*-core and total output, making it particularly informative when researchers have similar h-index values but substantially different publication volumes.

#### 2.3.2 Volume-normalized efficiency metric (he^*^)

Because the he-index is a ratio-based indicator, it is structurally sensitive to publication count, and comparisons across substantially different output levels may be distorted. To improve interpretability across varying publication volumes, a volume-normalized efficiency metric is additionally computed by benchmarking observed h-index values against the expected h-index conditional on publication count. The expected h-index is modeled using a power-law scaling function:


$$\begin{array}{l} ĥ=aN^{b}\; \end{array}$$

A normalized efficiency score is then defined as:


$$\begin{array}{l} he^{*}=\frac{h}{ĥ}\; \end{array}$$

Under this formulation, values of he^*^ > 1 indicate above-expected citation efficiency relative to typical performance at comparable publication volume, whereas he^*^ < 1 indicates below-expected efficiency. This normalized metric reduces the mechanical influence of publication count while preserving an efficiency-oriented interpretation.

### 2.4 External validation using FWCI

To strengthen evaluative relevance beyond *h*-derived indicators, FWCI was collected as an independent benchmark of field-normalized citation performance where available. FWCI values were extracted at the author level (overall portfolio average), ensuring consistency with the portfolio-level interpretation of the h-index and the proposed indicators. External validation was conducted by examining Spearman correlations between FWCI and both the raw he-index and the normalized efficiency score he^*^. FWCI values were available for 47 researchers; therefore, all FWCI-based analyses were conducted using this subset. FWCI was selected as an external benchmark because it is a widely used field-normalized citation indicator that accounts for disciplinary differences in citation practices, allowing comparison of citation performance across fields. As such, it provides an appropriate reference for evaluating whether the proposed normalized metric aligns with established measures of relative citation impact.

### 2.5 Statistical analysis

Given the skewed nature of bibliometric distributions, non-parametric methods were employed. Spearman's rank correlation coefficient (ρ) was used to examine monotonic relationships between (1) *he* and *N*, (2) *he* and *h*, and (3) he^*^ and *N*. To compare differences across groups, the Kruskal–Wallis test was used to evaluate whether the distribution of the he-index and normalized he^*^ differed significantly across career stages and across disciplinary domains. Descriptive statistics including mean, median, standard deviation, and range were also computed to support interpretation. Statistical significance was evaluated at the *p* < 0.05 level.

For the power-law normalization model, parameters were estimated using linear regression on log–log transformed data via ordinary least squares (OLS). Prior to model fitting, the data were visually inspected using scatter plots to identify potential outliers; no observations were excluded, as all values were considered representative of real-world bibliometric variation.

### 2.6 Ethical considerations

All data used in this study were derived from publicly accessible bibliographic profiles. Researchers' identities were anonymized in the analytical dataset, and findings are reported only at an aggregate level to ensure privacy and ethical compliance.

## 3 Results and discussion

To evaluate the empirical behavior and interpretability of the proposed he-index, a dataset of 54 researchers was compiled using publicly accessible Scopus author profiles. The sample covered three regions (USA, China, and Australia) and three academic domains (engineering, medicine, and social sciences). For each researcher, the total number of publications (*N*) and the h-index (*h*) were extracted, and the he-index was computed as:


$$\begin{array}{l} he=\left(\frac{h}{N}\right)\; \end{array}$$

As described in the methodology, the he-index is interpreted as an indicator of the proportion of publications represented by the h-index core, relative to the researcher's total publication output. However, because *he* = *h*/*N* is structurally sensitive to publication volume, a volume-normalized efficiency metric was additionally evaluated. The expected h-index conditional on publication count was estimated using a power-law scaling model:


$$\begin{array}{l} ĥ=1.02N^{0.7}\; \end{array}$$

And the normalized efficiency score was computed as:


$$\begin{array}{l} he^{*}=\frac{h}{ĥ}\; \end{array}$$

These parameters are empirically estimated from the present sample of 54 researchers and may vary across disciplines, datasets, or larger populations; therefore, recalibration may be required when applying the model in different contexts.

### 3.1 Descriptive trends across career stages

Descriptive statistics for the he-index and normalized efficiency metric (he^*^) across career stages are summarized in Table 2. The results show a clear decline in raw he-index values from early-career to senior researchers. Early-career researchers exhibited the highest mean he-index (mean = 0.50), followed by mid-career researchers (mean = 0.34) and senior researchers (mean = 0.24). This pattern suggests that early-career publication portfolios tend to be more compact, with a larger share of outputs contributing to the *h*-core, whereas senior portfolios typically expand over time, reducing the fraction of publications that actively support the h-index.

**Table 2:** Descriptive statistics of he-index and normalized efficiency (he^*^) by career stage.

Career stage	*n*	Mean (he)	SD	Median (he)	Mean (he^*^)	SD	Median (he^*^)
Early	18	0.50	0.22	0.50	0.94	0.40	0.98
Mid	18	0.34	0.12	0.31	1.11	0.27	1.13
Senior	18	0.24	0.12	0.24	1.18	0.40	1.34

Importantly, the normalized metric he^*^ yields a different interpretation. When citation efficiency is evaluated relative to the expected scaling relationship between *h* and *N*, differences across career stage become much weaker. This indicates that the apparent stage-level decline observed in the raw he-index is influenced substantially by the mathematical structure of the ratio *h*/*N* and the typical growth of *N* over time.

### 3.2 Structural dependence on publication volume

Correlation analysis confirms that the he-index is strongly dependent on publication volume. Spearman's rank correlation showed a strong negative association between he-index and total publications:


$$\begin{array}{l} \rho \left(he,N\right)=-0.767,\;p<0.001\; \end{array}$$

This relationship is expected for ratio-based indicators of the form *h*/*N*, since publication portfolios often grow faster than the h-index increases. Therefore, this negative association should not be interpreted as indicating lower academic contribution among highly productive researchers; rather, it reflects a structural sensitivity that may distort comparisons across researchers with widely different output volumes.

By contrast, the normalized metric he^*^ exhibited no significant relationship with publication count:


$$\begin{array}{l} \rho \left(he^{*},N\right)=0.064,p=0.645\; \end{array}$$

This indicates that normalization effectively removes the built-in penalty associated with publication volume and improves interpretability across different output scales.

### 3.3 Group comparisons across career stages and disciplines

Non-parametric tests were used to evaluate group-level differences due to the skewness typical of bibliometric indicators. A Kruskal–Wallis test confirmed that the raw he-index differs significantly across career-stage categories:


$$\begin{array}{l} H=16.086,p=0.0003\; \end{array}$$

However, after applying normalization, career-stage differences in he^*^ were no longer statistically significant:


$$\begin{array}{l} H=4.222,p=0.121\; \end{array}$$

This supports the interpretation that the strong stage-level differences in *he* are largely driven by the structural dependence of *h*/*N* on publication volume growth. Moreover, discipline-level comparisons indicated no significant differences in he-index across engineering, medicine, and social sciences (*p* = 0.179). Similarly, discipline differences in the normalized metric were not statistically significant (*p* = 0.317). These results suggest that within this dataset, publication-volume effects dominate disciplinary differences in shaping he-index values. The statistical results and correlation analysis for both metrics are summarized in Table 3.

**Table 3:** Group comparison tests and correlation analysis for he-index and he^*^.

Analysis	Metric	Test statistic	*p*-value	Interpretation
Career stage difference	He	Kruskal–Wallis (*H* = 16.086)	0.0003	Significant stage variation
Career stage difference	(he^*^)	Kruskal–Wallis (*H* = 4.222)	0.121	Not significant after normalization
Discipline difference	He	Kruskal–Wallis (*H* = 3.439)	0.179	Not significant
Discipline difference	(he^*^)	Kruskal–Wallis (*H* = 2.298)	0.317	Not significant
Output dependence	he vs. (*N*)	Spearman (rho = −0.767)	<0.001	Strong structural dependence
Output dependence	(he^*^) vs. (*N*)	Spearman (rho = 0.064)	0.645	Dependence removed

### 3.4 External validation using FWCI

To provide external validation using an independent field-normalized benchmark, FWCI values were available for 47 researchers. The raw he-index (*he* = *h*/*N*) showed no significant association with FWCI (Spearman ρ = −0.175, *p* = 0.238), indicating limited external alignment when interpreted as an unadjusted ratio. In contrast, the normalized efficiency metric he^*^ exhibited a significant positive association with FWCI (Spearman ρ = 0.419, *p* = 0.003). This result supports the evaluative relevance of the normalization approach and suggests that he^*^ captures an efficiency-oriented dimension of scholarly performance that aligns with recognized impact benchmarks beyond cumulative measures alone.

In addition to external validation using FWCI, the robustness of the proposed metrics is supported by multiple complementary validation approaches. These include (i) the independence of the normalized metric from publication volume, indicating effective normalization, (ii) its stability across career stages and disciplinary groups, and (iii) its ability to distinguish between researchers with similar h-index values but substantially different publication volumes, revealing differences in the concentration of impactful publications within their research portfolios. Together, these analyses constitute a multi-dimensional validation framework, demonstrating that the behavior of the proposed metrics is consistent across independent analytical dimensions, including independence from publication volume, stability across career stages and disciplines, and alignment with an external benchmark (FWCI), and is not solely dependent on a single validation approach.

### 3.5 Implications for research assessment

While widely used indicators such as the h-index and its variants provide a useful summary of cumulative research performance, they do not explicitly capture how citation impact is distributed across a researcher's publication portfolio, and remain inherently influenced by publication volume and career length. As noted in prior studies, cumulative metrics may assign similar scores to researchers despite differences in their underlying citation patterns, limiting their interpretability in comparative evaluation contexts (Bornmann and Daniel, 2008; Ioannidis et al., 2020; Sugimoto and Larivière, 2018; Waltman, 2016). In particular, they do not distinguish between impact that is concentrated within a relatively small number of influential publications and impact that is more diffusely distributed across a larger body of work. The proposed he-index addresses this limitation by quantifying the proportion of publications contributing to the *h*-core, thereby providing information on impact concentration that is not available from cumulative indicators alone.

The results highlight a key interpretive distinction between cumulative and concentration-based indicators. While the h-index reflects cumulative achievement, the he-index captures impact concentration, describing the fraction of publications supporting the *h*-core. This distinction may be helpful in contexts where evaluators wish to understand whether impact is achieved through a compact set of influential papers or through a larger portfolio with more dispersed contributions.

At the same time, the strong dependence of *he* = *h*/*N* on publication volume demonstrates that the raw he-index should not be used in isolation for cross-career ranking. The normalized metric he^*^ provides a more robust alternative by benchmarking citation performance against expected scaling behavior, enabling a fairer comparison across researchers with different output levels. This aligns with broader recommendations that bibliometric indicators should be interpreted contextually and used in combination rather than as deterministic ranking instruments (Hicks et al., 2015; Moed, 2005; Panaretos and Malesios, 2009; Sugimoto and Larivière, 2018; Waltman, 2016). In practical terms, these indicators can support more informed decision-making in academic hiring, promotion, and funding allocation by complementing traditional cumulative metrics with information on impact structure and efficiency. Rather than serving as standalone ranking tools, they provide additional dimensions for interpreting research performance, particularly in relation to how impact is distributed and how it scales with publication volume. Conversely, evaluation systems that rely solely on cumulative indicators may inadvertently favor quantity-driven publication strategies, potentially overlooking researchers with more efficient or concentrated impact profiles.

More specifically, the proposed indicators are particularly valuable in evaluation scenarios where candidates exhibit similar cumulative performance but differ in the structure of their citation impact. For example, in hiring or promotion decisions involving researchers with comparable h-index values, the he-index enables evaluators to distinguish between impact concentrated in a limited number of highly influential publications and impact distributed across a broader set of outputs. This distinction is especially relevant in contexts that prioritize breakthrough contributions over incremental productivity. In addition, the normalized metric can be used to reduce bias associated with publication volume, supporting fairer comparisons between early-career and senior researchers, or between individuals following different publication strategies. These targeted applications demonstrate the added value of the proposed framework beyond conventional cumulative indicators.

To further illustrate the practical interpretability and added value of the proposed indicators, empirical examples based on real researchers from the dataset (see Supplementary material) are presented in Table 4. In the first case (Case A), two researchers in the medical field (R45 and R46) exhibit identical h-index values but differ in their publication outputs, resulting in distinct he-index values. While the h-index suggests equivalent cumulative impact, the he-index reveals that R46 achieves this impact through a higher concentration of impactful publications relative to its total output. In other words, a larger proportion of R46's publications contribute to the *h*-core compared to R45. This distinction is further reflected in the normalized metric (he^*^), where R46 demonstrates slightly higher efficiency relative to expected performance given its publication volume.

**Table 4:** Illustrative empirical comparison of researchers with similar h-index values, highlighting differences in he, he^*^, and FWCI.

Case	ID	Field	*N*	h-index	he (%)	he^*^	FWCI
A	R45	Medical	39	17	43.6	1.3	1.18
A	R46	Medical	34	17	50.0	1.4	1.3
B	R39	Engineering	85	23	27.0	1	1.84
B	R40	Engineering	107	24	22.0	0.9	1.03

In the second case (Case B), two researchers in engineering (R39 and R40) have comparable h-index values but differ more substantially in publication output. Accordingly, their he-index values indicate that R40's impact is more dispersed across its publication portfolio, whereas R39 exhibits a relatively higher concentration of impactful publications. The normalized metric (he^*^) further reinforces this contrast, with R39 slightly exceeding expected performance, while R40 falls marginally below expectation. Notably, these differences are also consistent with FWCI values, providing additional support for the interpretive validity of the proposed indicators in relation to established field-normalized metrics.

These examples demonstrate that, even among researchers with similar cumulative impact, the proposed indicators provide additional insight into both the concentration and efficiency of scholarly output, offering a more nuanced interpretation of research performance than the h-index alone.

### 3.6 Limitations and future directions

Several limitations should be considered. First, the sample size, while balanced and structured, remains modest for broad generalization, large bibliographic databases such as Scopus or Web of Science are well positioned to calibrate and refine the proposed normalization model using substantially larger and more diverse datasets, thereby improving generalizability and facilitating broader application in research evaluation systems. Second, the analysis relies on Scopus profiles and is subject to differences in database coverage and author disambiguation. Third, the study does not adjust for co-authorship patterns, self-citation behavior, or contribution roles, which can affect citation-based indicators (Põder, 2022).

Finally, although the normalized metric reduces dependence on publication volume, its evaluative relevance is supported by its significant positive association with the external benchmark of FWCI. This finding indicates that the normalized indicator aligns with established field-normalized measures of research impact. Further validation using additional external indicators, such as top percentile publications, competitive funding outcomes, or peer-review assessments, could provide complementary evidence in future work. Future research may also extend this analysis using larger samples and multiple bibliographic databases, while exploring discipline-specific scaling relationships and assessing robustness across different citation cultures.

## 4 Conclusion

This study introduced the he-index as a simple and interpretable bibliometric indicator designed to complement cumulative impact measures such as the h-index. Defined as *he* = (*h*/*N*), the he-index quantifies the proportion of a researcher's publication portfolio that contributes to the h-index structure, providing a transparent description of impact concentration (impact density) rather than total output. Using a structured dataset of 54 Scopus-indexed researchers across engineering, medicine, and social sciences from the USA, China, and Australia, the analysis demonstrated that the he-index varies systematically across publication profiles and is strongly influenced by total publication volume.

The results confirmed that the raw he-index exhibits a strong negative association with total publications, reflecting an expected structural property of ratio-based indicators. Therefore, while the he-index offers useful interpretive insight into portfolio concentration, it should not be used as a standalone ranking metric across researchers with substantially different output scales or career lengths. To address this limitation, the study proposed a volume-normalized efficiency metric, he^*^, derived by benchmarking the observed h-index against the expected h-index conditional on publication count using a power-law scaling model. The normalized metric substantially reduced dependence on publication volume, offering a more comparable efficiency-oriented indicator across researchers with different levels of output.

Overall, the findings support the view that research performance is multidimensional and cannot be adequately captured by a single cumulative indicator. The he-index and its normalized form he^*^ provide complementary information to traditional measures by distinguishing between cumulative achievement and the concentration of impact within a publication portfolio. In evaluation contexts such as academic promotion, recruitment, or grant assessment, these metrics may contribute to more nuanced interpretation when applied alongside established bibliometric indicators and informed qualitative review.

This study has limitations, including the modest sample size and reliance on a single bibliographic database. In addition, the indicators examined here do not account for co-authorship structure, self-citation behavior, or contribution roles, which may influence author-level citation measures. Future research should extend this work using larger, field-specific datasets, incorporate multiple bibliographic sources, and evaluate the relationship of the proposed metrics with independent benchmarks of research quality and contribution.

## Data Availability

The raw data supporting the conclusions of this article will be made available by the authors, without undue reservation.
