# Supplementary material for: Beyond cumulative impact: the he-index and a volume-normalized efficiency metric for research evaluation
Source: Front Res Metr Anal. 2026 May 4;11:1793664. doi: 10.3389/frma.2026.1793664 (PMC13180727; doi:10.3389/frma.2026.1793664)
Supplement: Supplementary file 1 [file Data_Sheet_1.docx]

**Table A1**. Anonymized dataset used in the empirical analysis

| **ID** | **Field** | **Region** | **N (Publications)** | **h-index** | **he (%)** | **FWCI** |
| --- | --- | --- | --- | --- | --- | --- |
| R1 | Engineering | USA | 1482 | 138 | 9.3 | 3.47 |
| R2 | Engineering | USA | 104 | 43 | 41.3 | 1.47 |
| R3 | Engineering | USA | 39 | 12 | 30.7 | NA |
| R4 | Engineering | USA | 50 | 13 | 26 | NA |
| R5 | Engineering | USA | 16 | 4 | 25 | NA |
| R6 | Engineering | USA | 15 | 7 | 46.6 | NA |
| R7 | Medical | USA | 463 | 105 | 22.6 | 2.99 |
| R8 | Medical | USA | 268 | 62 | 23 | 1.16 |
| R9 | Medical | USA | 96 | 28 | 29.2 | 1.05 |
| R10 | Medical | USA | 109 | 25 | 23 | 2.48 |
| R11 | Medical | USA | 36 | 4 | 11 | NA |
| R12 | Medical | USA | 4 | 2 | 50 | 0.17 |
| R13 | Social | USA | 189 | 52 | 27.5 | 2.2 |
| R14 | Social | USA | 110 | 36 | 32.7 | 3.7 |
| R15 | Social | USA | 50 | 20 | 40 | 0.97 |
| R16 | Social | USA | 26 | 11 | 42.3 | 0.87 |
| R17 | Social | USA | 35 | 3 | 8.6 | 0.09 |
| R18 | Social | USA | 6 | 5 | 83.3 | NA |
| R19 | Engineering | China | 982 | 43 | 4.4 | 0.94 |
| R20 | Engineering | China | 675 | 54 | 8 | 1.35 |
| R21 | Engineering | China | 86 | 16 | 18.6 | 0.63 |
| R22 | Engineering | China | 74 | 23 | 31.1 | 1.09 |
| R23 | Engineering | China | 9 | 6 | 66.6 | 1.18 |
| R24 | Engineering | China | 9 | 6 | 66.6 | 0.51 |
| R25 | Medical | China | 432 | 54 | 12.5 | 1.87 |
| R26 | Medical | China | 305 | 52 | 17 | 1.63 |
| R27 | Medical | China | 46 | 17 | 37 | 0.87 |
| R28 | Medical | China | 49 | 19 | 38.8 | 1.12 |
| R29 | Medical | China | 12 | 6 | 50 | 0.64 |
| R30 | Medical | China | 12 | 8 | 66.6 | 0.57 |
| R31 | Social | China | 82 | 32 | 39 | 2.52 |
| R32 | Social | China | 87 | 32 | 36.8 | 2.6 |
| R33 | Social | China | 50 | 26 | 52 | 2.24 |
| R34 | Social | China | 51 | 11 | 21.6 | 0.84 |
| R35 | Social | China | 6 | 4 | 66.6 | 1.47 |
| R36 | Social | China | 2 | 1 | 50 | 0.19 |
| R37 | Engineering | Australia | 397 | 74 | 18.6 | 2.77 |
| R38 | Engineering | Australia | 218 | 67 | 30.7 | 2.16 |
| R39 | Engineering | Australia | 85 | 23 | 27 | 1.84 |
| R40 | Engineering | Australia | 107 | 24 | 22 | 1.03 |
| R41 | Engineering | Australia | 10 | 3 | 30 | 9.76 |
| R42 | Engineering | Australia | 13 | 10 | 77 | 7.67 |
| R43 | Medical | Australia | 626 | 64 | 10 | 0.95 |
| R44 | Medical | Australia | 158 | 58 | 36.7 | 2.03 |
| R45 | Medical | Australia | 39 | 17 | 43.6 | 1.18 |
| R46 | Medical | Australia | 34 | 17 | 50 | 1.3 |
| R47 | Medical | Australia | 12 | 4 | 33.3 | 0.7 |
| R48 | Medical | Australia | 21 | 9 | 42.8 | 2.01 |
| R49 | Social | Australia | 197 | 54 | 27.4 | 2.9 |
| R50 | Social | Australia | 266 | 68 | 25.5 | 5.61 |
| R51 | Social | Australia | 60 | 15 | 25 | 0.82 |
| R52 | Social | Australia | 20 | 12 | 60 | 0.64 |
| R53 | Social | Australia | 3 | 2 | 66.6 | NA |
| R54 | Social | Australia | 3 | 2 | 66.6 | 0.64 |


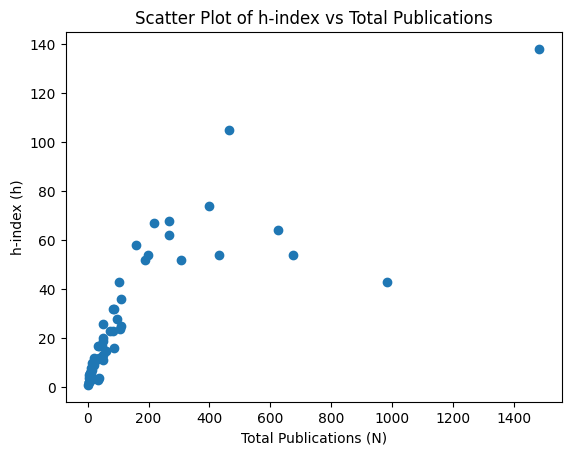


**Figure A1**. Scatter plot: h vs N

**
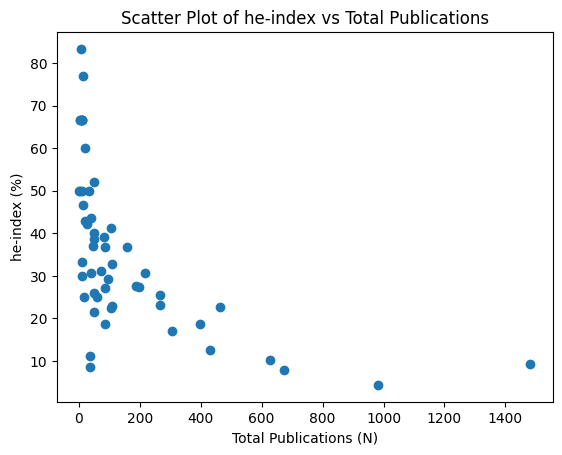
**

**Figure A2**. Scatter plot: he vs N


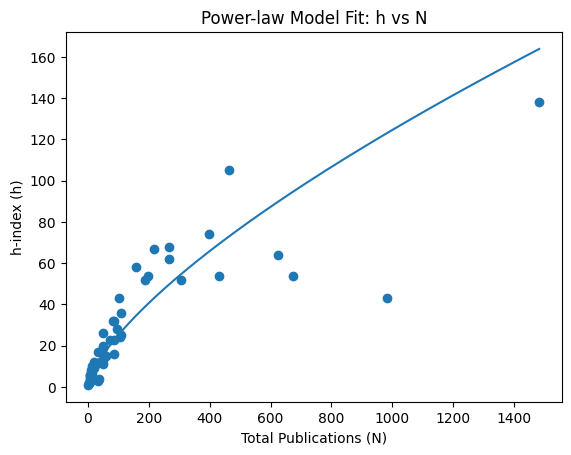


**Figure A3**. Power-law model fit (h vs N with fitted curve)


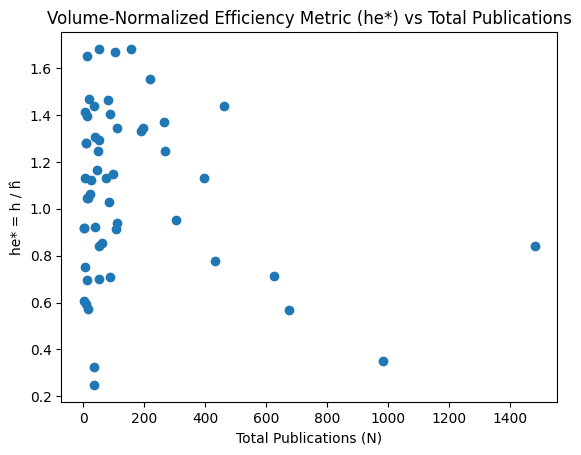


**Figure A4**. he* vs N scatter plot
